# *Bacillus anthracis* Lethal Toxin Disrupts TCR Signaling in CD1d-Restricted NKT Cells Leading to Functional Anergy

**DOI:** 10.1371/journal.ppat.1000588

**Published:** 2009-09-25

**Authors:** Sunil K. Joshi, Gillian A. Lang, Jason L. Larabee, T. Scott Devera, Lindsay M. Aye, Hemangi B. Shah, Jimmy D. Ballard, Mark L. Lang

**Affiliations:** Department of Microbiology and Immunology, University of Oklahoma Health Sciences Center, Oklahoma City, Oklahoma, United States of America; The Salk Institute for Biological Studies, United States of America

## Abstract

Exogenous CD1d-binding glycolipid (α-Galactosylceramide, α-GC) stimulates TCR signaling and activation of type-1 natural killer–like T (NKT) cells. Activated NKT cells play a central role in the regulation of adaptive and protective immune responses against pathogens and tumors. In the present study, we tested the effect of *Bacillus anthracis* lethal toxin (LT) on NKT cells both *in vivo* and *in vitro*. LT is a binary toxin known to suppress host immune responses during anthrax disease and intoxicates cells by protective antigen (PA)-mediated intracellular delivery of lethal factor (LF), a potent metalloprotease. We observed that NKT cells expressed anthrax toxin receptors (CMG-2 and TEM-8) and bound more PA than other immune cell types. A sub-lethal dose of LT administered *in vivo* in C57BL/6 mice decreased expression of the activation receptor NKG2D by NKT cells but not by NK cells. The *in vivo* administration of LT led to decreased TCR-induced cytokine secretion but did not affect TCR expression. Further analysis revealed LT-dependent inhibition of TCR-stimulated MAP kinase signaling in NKT cells attributable to LT cleavage of the MAP kinase kinase MEK-2. We propose that *Bacillus anthracis*–derived LT causes a novel form of functional anergy in NKT cells and therefore has potential for contributing to immune evasion by the pathogen.

## Author Summary

The bacterium *Bacillus anthracis* is the causative agent of anthrax infection. Anthrax is a life-threatening disease caused by inhalation or ingestion of spores, or transmission through wounds and abrasions. The bacterium secretes toxins, proteins that enter numerous cell types in an infected individual altering their function and contributing to the disease process. Consequently, we and other researchers are dedicated to identifying cells affected by anthrax toxins and to understanding the implications for the function of those cells. We have observed that anthrax toxins adversely affect a type of cell in the immune system known as a natural killer–like T (NKT) cell. NKT cells are found in the bone marrow, blood, spleen, lymph nodes and liver and are required for optimal immune responses. We report that anthrax toxins effectively shut down NKT cells, preventing them from functioning normally. We propose that this has serious consequences because anyone infected with *Bacillus anthracis* will be less able to mount an immune response against it.

## Introduction

CD1d-restricted Type I NKT cells are central regulators of adaptive immune responses [1]. NKT cells can be activated with exogenous CD1d-binding glycolipid antigen (Ag) to boost humoral immunity and prime CTL responses specific for co-administered Ag [2],[3],[4]. The mechanisms by which NKT cells enhance antibody (Ab) responses are unclear but involve cognate and non-cognate interactions between NKT cells and B cells [5],[6],[7]. The interactions of dendritic cells (DC) and NKT cells have been studied more extensively. NKT cells can drive DC maturation and induction of tumor-specific CTL [8],[9], a mechanism likely important for NKT-enhanced tumor rejection [4],[10]. There is also evidence for increased priming of Th responses as a result of NKT activation suggesting an important contribution to the initiation of adaptive immunity [4].

NKT cells contribute to the adaptive (and protective) immune response to bacterial, viral and potentially parasitic infections [11],[12],[13]. However, the effects of pathogen-derived virulence factors including bacterial exotoxins on NKT function have received little attention. The Bovine Calmette-Guerin (BCG) bacterium, bacterial LPS and CD1d-binding glycolipids can induce a state of non-responsiveness or anergy in NKT cells although it is not appreciated whether this is reflective of pathogen-derived products subverting the host immune response and/or the host limiting the production of tissue-damaging NKT products following infection [14],[15].

*Bacillus anthracis* lethal toxin (LT) is a paradigm for virulence factors that strongly subvert and suppress host immunity during disease [16],[17],[18]. Anthrax involves severe and extensive bacteremia with the organism growing to very high numbers (>10^8^ organisms/ml) in the bloodstream. This high level of growth is thought to be possible because LT severely cripples the host immune system. Thus, the mechanism by which LT intoxicates cells has been the subject of intense study. In order to disrupt target-cell function, protective antigen (PA) interacts with specific cell surface receptors, forms a heptameric structure that is bound by LF, and the complex is endocytosed into the cytosol [19]. Once delivered by PA into the cell, LF cleaves and inactivates MAPKKs, such as MEK-2, leading to disruption in MAPK signaling [20]. Yet, despite these many important advances in understanding the mechanism of cellular intoxication by LT, little is known about the specific types of immune cells targeted by this toxin during disease.

In the present study, we tested the hypothesis that a sub-lethal dose of LT administered *in vivo* could impact murine NKT cell function. We report that NKT cells express anthrax toxin receptors and bind PA to a greater degree than other types of immune cell. LT also causes reduced expression of several activation-inducing molecules on NKT cells. Furthermore, LT treatment inhibited NKT activation by CD1d/glycolipid complexes as evidenced by reduced cytokine secretion. These results indicate that one of the immune evasion/suppression strategies employed by *B. anthracis* involves reduced NKT activation.

## Materials and Methods

### Toxin expression and purification

Histidine-tagged PA and LF were expressed separately in competent BL21 *Escherichia coli* (Invitrogen, Carlsbad, CA) transformed with the pET15b-rPA, pET15b-rLF and pET15brLF^H690C^ plasmids respectively (gifts from Dr. J. Collier, Harvard Medical School). PA and LF were then purified from bacterial lysates using standard methods also described previously [21]. In brief, PA and LF purification was achieved using a 5 ml HisTrap FF affinity column (GE LifeSciences, Piscataway, NJ). LPS contamination was removed by from purified PA and LF using an EndoTrap Red LPS-binding affinity column (Lonza, Walkersville, MD). The concentration of residual endotoxin was determined using the Limulus Assay, which has a sensitivity of 0.06 Endotoxin Units/ml (6 pg/ml) (Lonza). The final preparation used for this study contained 40 ng/ml LPS in the PA and was below the limits of detection for the LF. No more than 4 ng LPS was administered to each mouse in experiments described herein.

### Mice

Female C57BL/6 mice were purchased from the National Cancer Institute (Bethesda, MD) and used for experiments at 6–10 wk of age. Jα18^−/−^ mice (also known as Jα281^−/−^) on the C57BL/6 genetic background were obtained from Dr. M. Kronenberg (La Jolla Institute for Allergy and Immunology) with kind permission from Dr M. Taniguchi (RIKEN Institute, Japan). The Institutional Animal Care and Use Committee at the University of Oklahoma Health Sciences Center approved all procedures described herein.

### Toxin administration

200 µg of lethal toxin (1∶1 molar ratio of PA and LF) in sterile endotoxin-free PBS was administered *i.v.* by para-orbital injection whilst mice were anesthetized using 4% vaporized isofluorane. Mice were euthanized 4 days after toxin injection. Spleen, liver, thymus and LN were harvested.

### Cell isolation

Single cell suspensions were prepared from the harvested tissue by mechanical disruption. Erythrocytes were removed by lysis with ammonium chloride buffer (0.16 M NH_4_Cl, 0.17 M Tris- HCl, pH 7.4). Cells were washed in RPMI 1640 and cell number and viability were assessed by trypan blue exclusion. Hepatic lymphocytes were obtained from hepatocytes by percoll density gradient centrifugation.

### Antibodies and fluorochromes

Allophycocyanin (APC-) or PE-conjugated CD1d tetramer loaded with α-GC was obtained from the NIAID tetramer facility (Emory University, Atlanta, GA). 2.4G2 Fc-blocking mAb was purchased from BioXcell (Lebanon, NH). Anti-CD49b, -CD3, -CD28 and -TCRβ mAbs were purchased from BD Biosciences (Palo Alto, CA). Anti-Erk1/2 and anti-phospho-Erk1/2 polyclonal Abs were purchased from Cell Signaling Technology (Worcester, MA). Anti-MEK-2 N-terminal polyclonal Ab was purchased from Santa Cruz Biotechnology (Santa Cruz, CA). Anti-GAPDH mAb was purchased from Abcam Inc. (Cambridge, MA). All other antibodies were from e-Bioscience (San Diego, CA). Alexa 647-conjugated PA was produced in house using an Alexa labeling kit according to manufacturer's instructions (Invitrogen, Carlsbad, CA).

### Flow cytometry

The 2.4G2 mAb was added to cell suspensions at a final concentration of 20 µg/ml. Cells were then incubated at room temperature for 1 h with a 1∶250 dilution of CD1d tetramer and appropriate mAbs at a 1∶100 −200 dilution. Unbound mAbs and tetramer were removed by washing cells three times in PBS. Cells were fixed with 1% v/v p-formaldehyde in PBS and samples were then analyzed by flow cytometry (FACSCalibur, Becton Dickinson, Palo Alto, CA.).

### *In vitro* NKT stimulation

Four similar approaches were taken to stimulate NKT cell TCR signaling. (i) Splenocytes were obtained from mice treated with LT *in vivo*. Splenocytes were then stimulated *in vitro* with α-GC (BioMol, Cambridge, MA) at a final concentration of 50 ng/ml. After 24 and 48 h, supernatants were collected and stored at −80°C. (ii) Splenocytes were obtained from NKT-deficient Jα18^−/−^ mice and *in vitro*-expanded NKT cells were obtained from splenocytes of C57BL/6 mice using a modified version of the method reported by the Taniguchi laboratory [22]. NKT cell purity as defined by CD1d tetramer binding was 85%–96% in the experiments reported herein. Jα18^−/−^ splenocytes and C57BL/6 NKT cells were treated for 1 h with PBS or LT at a final concentration of 1 µg/ml. After washing the PBS-treated and LT-treated cells were cultured separately or co-cultured (splenocytes plus NKT cells) in the absence or presence of α-GC at a final concentration of 50 ng/ml for 48 h. The supernatants were then collected and stored at −80°C until required. IL-4 and IFNγ concentrations in the supernatants were then determined by Multi-Plex cytokine analysis according to manufacturer's instructions using the Bio-Plex assay system (Bio-Rad, Hercules, CA). (iii) Anti-NK1.1-enriched NKT cells (52% pure) were treated with PBS or LT *in vitro* and then cultured on 96 well plates coated with anti-CD3 mAb at a coating concentration of 10 µg/ml and with media containing 10 µg/ml anti-CD28 mAb. After 48 h, cell culture supernatants were collected for analysis. In these assays, the remaining cells were tetramer^−^/TCR^+^/NK1.1^+^ cells, NK cells and there were no detectable T cells (iv) Anti-NK1.1-enriched NKT cells were treated with PBS or LT and then coated with anti-CD3 mAb at 4°C before washing and cross-linking the bound Ab by addition of an anti-Armenian Hamster IgG solution (Rockland Inc., Rockland, MD) at 37°C. After 5 minutes cells were pelleted by a 7 s micro-centrifuge pulse. Pellets were immediately re-suspended in ice-cold lysis buffer containing: 1.0% v/v NP40; a protease inhibitor cocktail (Roche Systems Inc., Pleasanton, California); 4.0 mM Na_3_VO_4_ and 2.0 mM NaF. After incubation on ice for 30 min, lysates were then clarified by centrifugation at 13,000 RCF for 10 min at 4°C. Supernatants were collected and stored at −20°C until required.

### Immunoblot

Cell lysates were subjected to SDS-PAGE under reducing conditions and proteins were then transferred to nitrocellulose membranes using a Bio-Rad semi-dry blotter. Membranes were blocked with 5% w/v non-fat dry milk in PBS containing 0.5% v/v TWEEN-20 for 1 h before addition of primary Ab at a 1 µg/ml final concentration and incubation overnight at 4°C. Membranes were washed 6 times in PBS before incubation with HRP-conjugated secondary Ab in 3% milk in PBS containing 0.05% v/v TWEEN-20 and further washing. ECL Plus reagent (GE Healthcare, Piscataway, NJ) was used to detect membrane-bound Ab and imaged using Blue Basic Autoradiography Film (ISC BioExpress, Kaysville, UT).

## Results

### Protective antigen targets NKT cells

**Figure 1:**
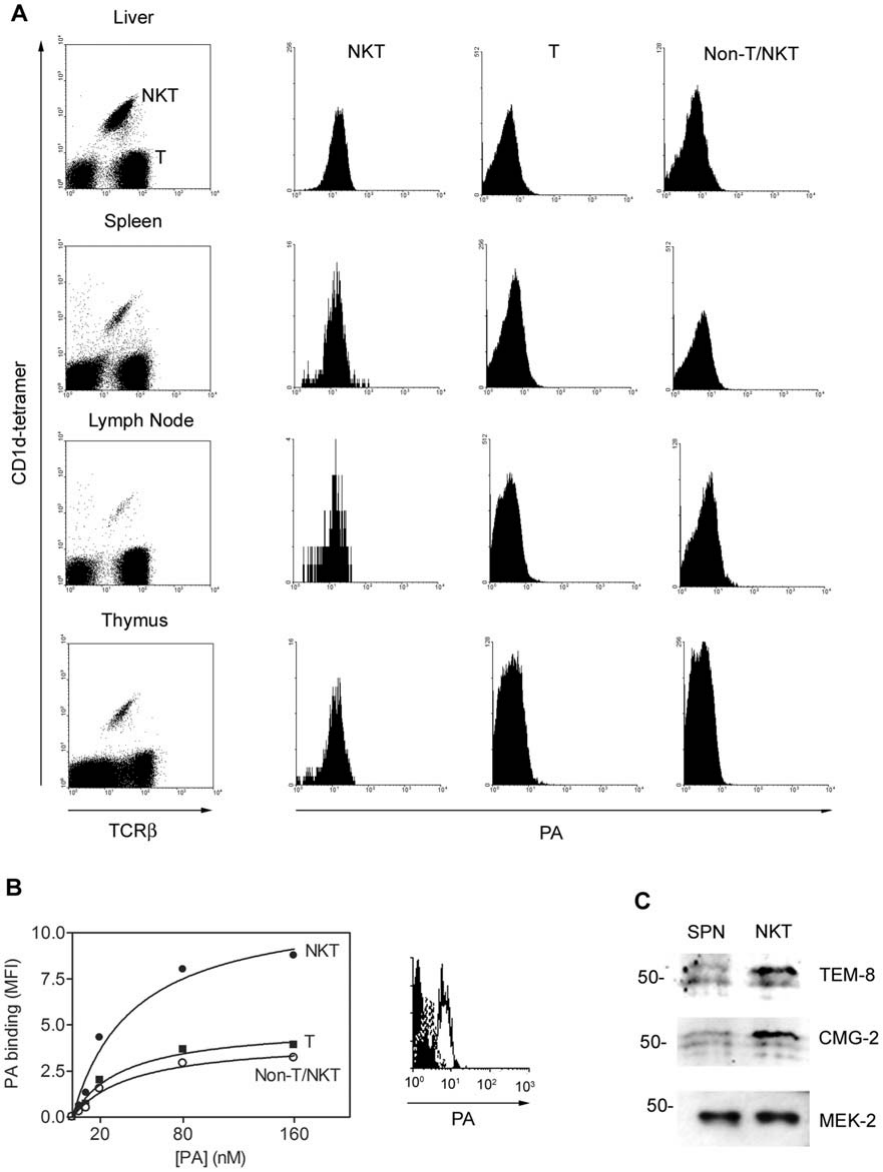
PA binds to NKT cells. Hepatic lymphocytes, splenocytes, lymph node cells and thymocytes were obtained from C57BL/6 mice. A Cells were stained with α-GC-loaded PE-CD1d tetramer and FITC-anti-TCRβ mAb to distinguish NKT cells, T cells and non-T/NKT cells. Samples were also counter-stained with Alexa647-PA to detect PA-binding proteins. Histograms show PA binding to each cell type. B Splenocytes were stained as described and with the concentrations of Alexa647-PA indicated. Graph shows effect of PA concentration on binding to different cell types. The histogram shows the effect of unlabeled PA on binding of Alexa647-PA. Filled histogram = no PA, continuous line = Alexa647-PA, dotted line = Alexa647-PA plus unlabeled PA. The data in A for spleen, lymph node and thymus are representative of three independent experiments. Binding of PA to hepatocytes in A and the dose response curve in B are from a single experiment. C Cell lysates were prepared from whole splenocytes (left lane) and hepatocytes (middle lane) of C57BL/6 mice (middle lane). NKT cells were enriched from the spleens of C57BL/6 mice and expanded *in vitro* for 5 d until 96% pure (right lane). Cell lysates were resolved by SDS-PAGE and examined by immunoblotting for the anthrax toxin receptors TEM-8 and CMG-2 and the anthrax toxin intracellular target MEK-2 which serves as a loading control.

Hepatic lymphocytes, splenocytes, lymph node cells and thymocytes were obtained from C57BL/6 mice. The cells were stained with α-GC-loaded CD1d-tetramer and anti-TCRβ mAb to distinguish between type I NKT cells (α-GC/CD1d tetramer^+^/TCRβ^+^), T cells (α-GC/CD1d tetramer^−^/TCRβ^+^) and non-NKT/T cells (α-GC/CD1d tetramer^−^/TCRβ^−^) and counter-stained with Alexa647-conjugated PA (Figure 1A). We observed that PA bound all α-GC/tetramer/TCRβ^+^ cells and did so to a greater extent than TCRβ^+^ T cells and non-T/NKT cells represented mainly by B cells and dendritic cells. The binding of PA to all cells could be saturated and then competed out with a 10-fold molar excess of unlabeled PA indicating expression of specific receptors (Figure 1B). The Kd for binding of PA to NKT cells was approximately 20 nM which is consistent with the observed expression of both known PA receptors Tumor Endothelial Marker-8 (TEM-8) and Capillary Morphogenesis protein-2 (CMG-2) with reported affinities *in vitro* of 130 nM and 0.17 nM respectively [23]. Purified splenic NKT cells were shown by immunoblot to express the 60 kDa and 45 kDa splice variants of TEM-8 and the 55 kDa form of CMG-2 (Figure 1C) at a greater abundance, relative to splenocyte lysates. The stronger signal obtained with the NKT lysates as compared to the splenic lysates is consistent with the differential expression detected by flow cytometry. These data show that NKT cells express higher amounts of the PA receptors than other hematopoietic cell types, suggesting NKT cells may be more susceptible to the effects of LT than other cell types.

### Lethal toxin alters expression of several NKT cell surface markers

**Figure 2:**
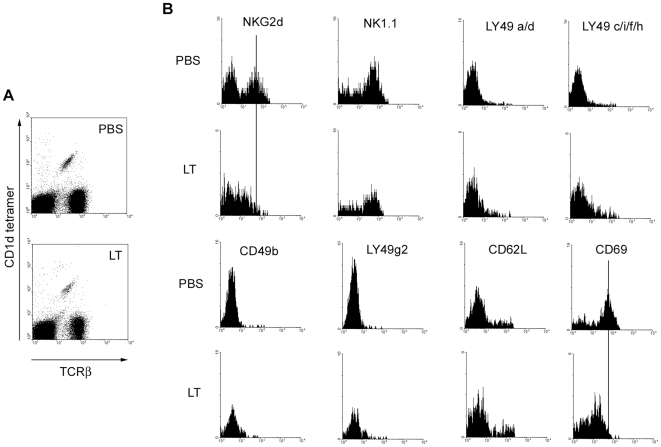
LT alters expression of selected NKT cell surface markers. C57BL/6 were treated with 100 µg of LT in PBS by the *i.v.* route or mock-treated with PBS alone. After 96 h, splenocytes were obtained and incubated with FcR-blocking mAb 2.4G2 in the presence of α-GC/CD1d tetramer, anti-TCRβ mAb and mAbs as indicated. Cells were then washed, fixed and analyzed by flow cytometry. A Shows α-GC/CD1d tetramer^+^/TCRβ^+^ NKT cells B Histograms show expression of indicated markers after gating on NKT cells. Data are representative of 3 independent experiments.

C57BL/6 mice were treated with a sub-lethal dose of LT and splenocytes were harvested 4 d later (Figure 2). We observed that LT treatment had no effect on binding of α-GC/CD1d tetramers or anti-TCRβ mAb, indicating that expression of the invariant Vα14/Jα18 TCR was not affected by LT. Similarly, TCRβ expression on T cells was not affected (Figure 2A). LT induced considerable splenomegaly increasing the total cellular yield approximately two fold but had only a small effect on the frequency (25% increase) of NKT cells in the spleen (Figure S1). The increase in splenic NKT cell numbers could be caused by recruitment from other locations, proliferation of splenic NKT cells or a combination of both effects.

Several NK- and NKT-associated activating and inhibitory receptors as well as other markers of NKT activation were then examined (Figure 2B). LT treatment resulted in decreased expression of the activation molecule NKG2d but had no effect on NK1.1 expression. The effect on NKG2D expression was specific to NKT cells since toxin did not alter NKG2D expression by NK cells in C57Bl/6 or Jα18^−/−^ mice (Figure S2A). Furthermore, active LT was required since a function-deficient mutant LT^H690C^ did not affect NKG2D expression by NKT cells (Figure S2B). A minor increase in the expression of the inhibitory LY49 receptors was observed following treatment with active LT (Figure 2B). There was also a small increase in the expression of CD62L and a substantial decrease in the expression of CD69 (Figure 2B). NKT cells have an innate ‘memory/activated phenotype’ and unlike naïve T cells (CD69^lo^/CD62L^hi^) are constitutively CD69^hi^CD62L^lo^ [24]. Changes in NKG2D expression by splenic NKT cells were not observed 3 h, 6 h and 14 d after LT *in vivo* administration (Figure S3A). In contrast, although splenocytes had returned to the pre-treatment phenotype by 14 d, LN cells still had reduced NKG2D expression (Figure S3B). Thymic NKT cells were also largely NKG2D^+^ indicating that the primary target of the toxin was NKT cells in the periphery rather than developing NKT cells in the thymus (Figure S3C). We also tested the effect of the toxin components PA and LF individually and did not observe splenomegaly, changes in NKT frequency, tetramer binding, or expression of TCRβ and NKG2D (*data not shown*). In further controls we determined that 20 ng, and 4 µg of LPS administered by the i.v. route did not result in reduced NKG2D or CD69 expression by NKT cells at 96 h as observed for LT (*data not shown*). This demonstrates that any residual LPS (estimated at no more than 4 ng per dose) in the LT preparation does not account for the observed effects of LT on NKT cells.

The overall effect of LT treatment therefore appears to be a shift from the memory/activated phenotype to that of an anergic NKT cell as evidenced by loss of NKG2D and CD69. In contrast, another study showed that LPS-induced anergy does not involve loss of CD69 by NKT cells and NKG2D expression was not examined [15]. However to the best of our knowledge these findings are the first to demonstrate that a bacterial exotoxin can alter the activation potential of NKT cells and it seems the mechanism is different from that employed by LPS.

### NKT cells from toxin-treated mice are viable and non-apoptotic

**Figure 3:**
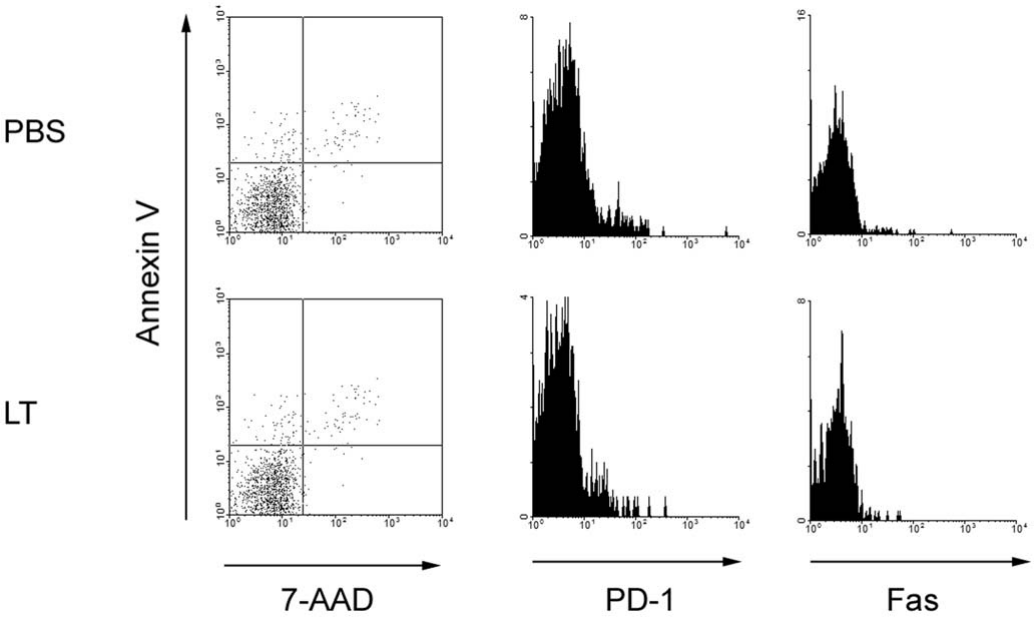
NKT cells from toxin-treated mice are viable and non-apoptotic. C57BL/6 were treated with 100 µg of LT in PBS by the *i.v.* route or mock-treated with PBS alone. After 4 d, splenocytes were obtained and incubated with FcR-blocking mAb 2.4G2 in the presence of CD1d tetramer and anti-TCRβ mAb and mAb or reagents indicated. After washing and fixation, cells were analyzed by flow cytometry. Data are representative of 3 independent experiments and show uptake of 7-AAD and expression of Annexin V, PD-1 and Fas by α-GC/CD1d tetramer^+^/TCRβ^+^ cells.

We assessed Annexin V versus 7-AAD (7-amino-actinomycin D) staining in α-GC/CD1d tetramer^+^/TCRβ^+^ NKT cells which allows one to distinguish live, non-apoptotic cells (AnnV^−^/7-AAD^−^) from early apoptotic (AnnV^+^/7-AAD^−^), late apoptotic (AnnV^+^/7-AAD^+^) and necrotic (AnnV^−^/7-AAD^+^) cells (Figure 3, left panels). We did not observe any changes in binding of Annexin V or uptake of 7-AAD by NKT cells following toxin treatment. We examined expression of the programmed-death-1 molecule (PD-1) recently reported to be required for NKT anergy induction [25]. LT treatment did not affect NKT expression of PD-1 (Figure 3, middle panels). We also assessed expression of Fas which was not altered by treatment with the toxin (Figure 3, right panels). This is in contrast to BCG which induces Fas up-regulation and causes NKT non-responsiveness to antigenic stimulation [15]. These results suggest that the sub-lethal dose of toxin administered in our study does not result in cell death, apoptosis or PD-1-dependent anergy in NKT cells and reinforces the concept that the mechanisms of anergy induction by BCG, LPS, α-GC and LT are different from each other.

It is possible that our method of gating on live lymphocytes by FSC/SSC profile excludes cells that have been killed by LT. We therefore gated on FSC^lo^/SSC^hi^ cells and assessed the effect of toxin on the number of ‘dead’ cells as well as the frequency of α-GC/CD1d tetramer-binding cells and their Annexin V binding and 7-AAD uptake (Figure S4). LT had very little effect on the number of non-viable cells recovered but there was considerable non-specific binding of tetramer to those dead cells. As expected those cells were either AnnV^+^/7-AAD^+^ or AnnV^−^/7-AAD^+^ and thus non-viable. Therefore while LT is likely to result in NKT necrosis *in vivo*, those cells are likely cleared from the spleen and live non-apoptotic cells are recovered in our assays.

### LT inhibits CD1d/α-GC-stimulated cytokine production

**Figure 4:**
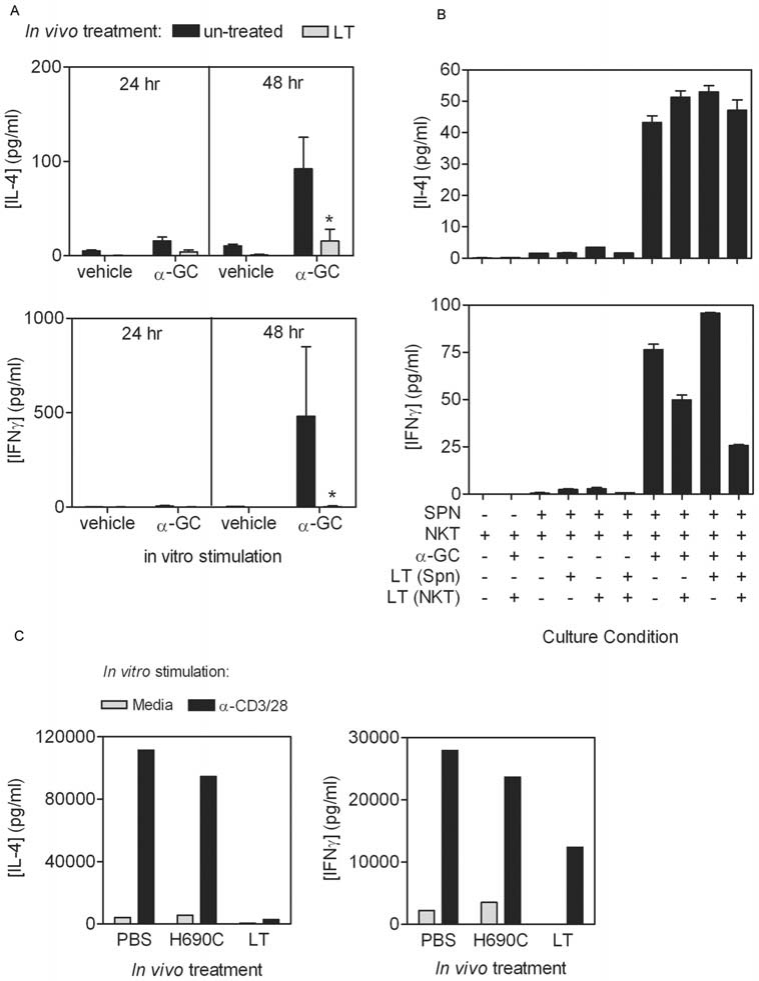
LT inhibits α-GC-stimulated cytokine production. A C57BL/6 mice were treated with 100 µg of LT in PBS by the i.v. route or mock-treated with PBS alone. After 4 d, splenocytes were obtained and stimulated *in vitro* with α-GC at a final concentration of 50 ng/ml. Supernatants were collected after a further 24 and 48 h and stored at −80°C. B Splenocytes from Jα18^−/−^ mice and *ex vivo*-expanded NKT cells from C57BL/6 mice were treated *in vitro* with LT at a final concentration of 1 µg/ml for 1 h before washing and culturing separately or together in the presence or absence of α-GC as described in A. IL-4 and IFNγ concentrations in the supernatants were then determined by Bio-Plex analysis. Data show mean cytokine concentration for 3 mice per group ±SD. Asterisk indicates significant difference between cytokine concentration in un-treated control and samples from LT-treated mice. C C57BL/6 mice were treated with PBS, non-functional LT mutant, or wild type LT before enrichment of NKT cells using anti-NK1.1-based magnetic isolation. Cells were stimulated with anti-CD3 and CD28 mAbs and culture supernatants collected after 48 h.

LT induced an inactivated but not an apoptotic phenotype. We therefore determined if prior toxin treatment affected TCR-mediated NKT activation (Figure 4). We obtained splenocytes from PBS- and LT-treated mice and stimulated them *in vitro* with α-GC. We observed that LT-treated mice had a substantially reduced production of IL-4 and IFNγ following toxin treatment (Figure 4A). While this experiment showed that LT treatment reduced the ability of NKT cells to be activated by α-GC, it did not determine whether the LT was acting on APCs in the mixed splenocyte culture or the NKT cells or both. Published reports show that DCs, macrophages, B cells and Th cells can all be targeted by anthrax toxins [16],[17],[18],[26], and thus it is conceivable that the target of LT in these cultures is the APC. To address this issue we separately exposed the NKT cells and the APCs (Jα18^−/−^ splenocytes) to LT, washed the cells and then cultured them separately or together with and without α-GC (Figure 4B). For this approach we obtained splenocytes from Type I (α-GC-reactive) NKT-deficient Jα18^−/−^ mice and *in vitro*-expanded NKT cells derived from C57BL/6 splenocytes. We treated Jα18^−/−^ splenocytes and NKT cells with PBS or LT before washing and then co-cultured the cells with or without α-GC (Figure 4B). We observed that IL-4 production was not affected, but IFNγ production was inhibited when the NKT cells but not the Jα18^−/−^ splenocytes that were treated with LT. The reason for the lack of inhibition of IL-4 production in Figure 4B is not clear, but may be attributable to the behavior of the *in vitro*-expanded NKT cells as compared to the whole C57BL/6 splenocyte cultures. It is possible that the Th1/Th2 balance in ex vivo-expanded NKT cells is more readily disrupted following toxin treatment than in NKT cells in freshly harvested mixed splenocytes.

We also confirmed that the effect of LT on NKT cytokine production required active LF (Figure 4C). Mice were treated *in vivo* with PBS, the non-functional mutant PA+LF^H690C^, or active LT before enriching NKT cells and stimulating them *in vitro* with anti-CD3 and CD28 mAbs. We observed that only the active toxin was able to suppress cytokine production by the NKT cells. It should be noted that the method used for NKT stimulation in Figure 4C (CD3 and CD28 cross-linking rather than stimulation with α-GC), may have resulted in activation of Type I (α-GC-reactive) and Type II (α-GC-non-reactive) NKT cells. However, flow cytometric analysis revealed that 80–88% of the NK1.1^+^/TCRβ^+^ cells in the NKT preparation were α-GC/CD1d tetramer-binding and therefore Type I NKT cells. These data therefore show by different methods of NKT isolation, culture and stimulation, that LT has a direct effect on NKT function with respect to cytokine production.

### LT inhibits TCR signaling in NKT cells

**Figure 5:**
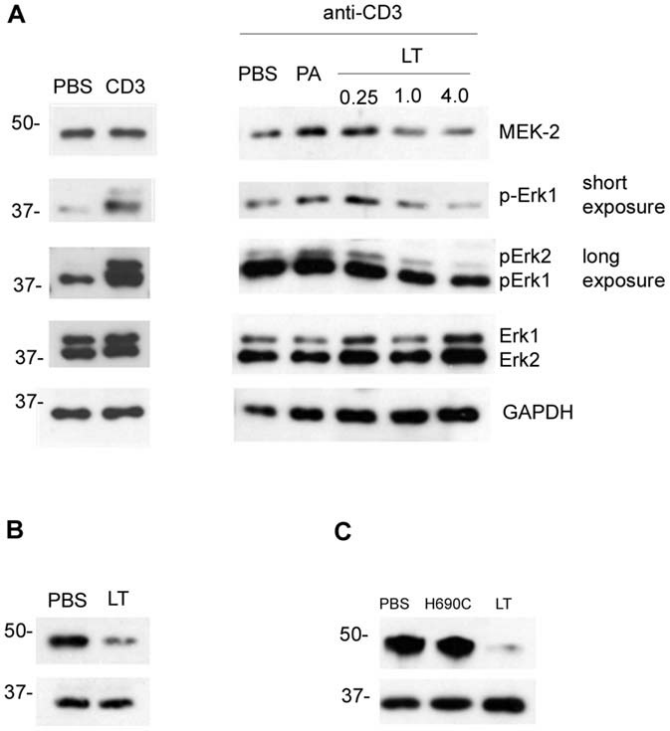
LT inhibits TCR-mediated signaling in NKT cells. A NKT cells were enriched from C57BL/6 splenocytes. Cells were then *in vitro* treated with PBS, PA (4 µg/ml) or LT (0.25 µg/ml of PA plus 0.25 µg/ml of LF), or 1.0 µg/ml or 4 µg/ml of each protein, and then stimulated by cross-linking CD3. Cell lysates were prepared and resolved by SDS-PAGE and transferred to nitrocellulose membrane before immunoblotting for MEK-2 N-terminus, phospho-Erk, total Erk, and the GAPDH loading control. Left panels show un-stimulated versus stimulated controls. Right panels show samples where CD3 was cross-linked. Two exposure times are indicated for phospho-Erk (short = 10 s, long = 90 s). B C57Bl/6 were treated with LT *in vivo* as described in materials and methods before collecting splenocytes and using CD1d-tetramers in conjunction with magnetic beads to obtain highly purified NKT cells. Lysates were prepared and subjected to SDS-PAGE and immunoblot analysis as indicated. C Experiment in B was repeated except that mice were treated with PBS, non-functional LT mutant, or wild type LT and NKT cells were obtained using the anti-NK1.1 enrichment method.

The MAPKK enzyme MEK-2 is proteolytically cleaved at the N-terminus by lethal factor that has gained entry to TEM-8- and CMG-2-expressing cells [20]. Phosphorylation of Erk (extracellular signal-regulated protein kinase) downstream of MEK-2 is a critical component of TCR-signaling-induced cytokine transcription [27]. We therefore performed experiments to determine if the lack of CD1d/α-GC-inducible cytokine was attributable to effects on the MAP Kinase/Erk signaling pathway. Enriched splenic NKT cells were treated with PBS or LT and then stimulated by CD3 cross-linking before preparation of cell lysates. Immunoblot analysis was conducted to examine the effect on MEK-2, and the status of TCR-induced Erk phosphorylation (Figure 5A). We used a mAb specific for the N-terminus of the MEK-2 protein which should have a decreased abundance in LT-treated cells. We observed that LT but not PA caused a loss of signal indicating N-terminal cleavage by LT. When NKT cells were treated with LT at three different doses, phosphorylation of Erk was diminished in a dose-dependent manner.

We also treated C57BL/6 mice with LT or PBS *in vivo* and then harvested splenocytes. NKT cells were isolated using α-GC-loaded CD1d tetramers and analyzed by SDS-PAGE and immunoblotting (Figure 5B). We observed that NKT cells recovered from LT-treated mice had a much lower amount of MEK-2 N-terminus than in NKT cells of PBS-treated mice. This demonstrates that LT leads to cleavage of NKT MEK-2 *in vivo*. We repeated this experiment, comparing the effects of PBS, the non-functional LT mutant and the wild type LT (Figure 5C). Consistent with our data in Figure 4, we observed that only the wild type LT resulted in cleavage of NKT MEK-2 *in vivo*.

## Discussion

We demonstrated for the first time that anthrax LT had adverse effects on NKT cell function. The mechanism of action involved cleavage of MEK-2 and reduced phosphorylation of Erk. This was evidenced by reduced TCR-stimulated cytokine production. Indeed, reduced MAP kinase activation including diminished phosphorylation of Erk is evident in anergic Th cells [28],[29], potentially explaining why LT induces anergy-like unresponsiveness in NKT cells stimulated via the TCR. We observed that both IFNγ and IL-4 production was refractory to LT, with IFNγ being inhibited to a greater extent when the TCR was directly engaged by CD1d/α-GC. This is consistent with published findings that there is a hierarchical requirement for TCR induced MAP kinase signaling in transcription of IFNγ and IL-4 genes by Th cells [30]. At present, it is not clear why we observe the clearest down-regulation of NKG2D expression at 96 h post toxin treatment and apparent recovery of expression by 14 d. Our data are likely reflective of the complex interplay of several factors including toxin delivery to NKT cells in secondary lymphoid organs; toxin entry and MEK-2 cleavage, reduced NKG2D transcription, degradation of existing NKG2D, the half life of LT *in vivo*, the lifespan of NKT cells and the rate of emigration of new NKT cells from the thymus during toxin-induced stress.

Although TCR-mediated activation of NKT cells was inhibited by anthrax LT, our data suggested that functional anergy may not be limited to TCR-stimulated responses. We observed a loss of NKG2D expression by NKT cells following LT treatment. The consistent down-regulation of NKG2D by NKT cells but not NK cells suggests that LT-treated NKT cells will be less responsive to ligation of killer-activatory receptors. At present, the implication for overall NKT function is not clear because information on the role of “NK receptors” on NKT cells is limited. NKG2D^+^ type II NKT cells (CD1d-restricted, non-reactive to ρ-GC) mediate damaging IFNγ-dependent immune responses to hepatitis B virus [31]. It is possible that the Type I NKT cells studied herein and expressing NKG2D have similar functions, leading to release of cytokines including IFNγ. It is also suggested that NKG2D could act as a TCR co-receptor and therefore be required for optimal cytokine production stimulated by CD1d/α-GC complexes [32]. The mechanism by which NKG2D expression is down-regulated is similarly unclear, but may be due to inhibition of the MAP Kinase pathway. Cross-linking of NK cell NKG2D leads to Erk phosphorylation and activation of MAP kinase-dependent cytotoxicity toward target cells [33]. It is possible that the MAP kinase signaling pathway governs expression of NKG2D by NKT cells. Further examination of this issue is warranted.

While anthrax LT can undoubtedly kill a proportion of a target cell population, in several studies, live toxin-treated cells are recovered and found to be functionally impaired [16],[17],[18]. This was observed for NKT cells whereby live, anergic cells were recovered that did not stain with Annexin V or with 7-AAD indicating that they were neither apoptotic or necrotic. Similarly, other indicators of apoptosis such as increased FAS expression were not detected. Most interesting, though is the observation that PD-1 was not increased on LT-treated NKT cells. PD-1 has been shown in a series of recent papers [25],[34],[35],[36] to be an important mediator of NKT anergy, whereby PD-1 blockade can prevent anergy or CD28 ligation can reverse the effects of α-GC-induced anergy [25],[36]. As indicated by our results, the anergy induced by LPS, α-GC and by anthrax LT may all be different, indicating that researchers will need to consider a broader array of NKT function in ascribing “anergy” to the functional status of an NKT cell.

Our data demonstrate that NKT cells are potentially a more significant target of the anthrax LT than other immune cell types because they express higher amounts of the TEM-8 and CMG-2 receptors (Figure 1). However, these data do not reveal if NKT cells are more significant targets during anthrax infection. We have observed that NKT cells and CD4^+^ T cells are similarly susceptible to LT *in vivo* and *in vitro* using the assays described herein (*data not shown*). This suggests the higher amounts of PA receptor on NKT cells as compared to CD4^+^ T cells may only determine susceptibility to LT when there are very low amounts of LT at the earliest stages of infection. Alternatively, CD4^+^ T cells and NKT cells may both express great enough numbers of PA receptors to allow MEK-2 cleavage sufficient to prevent TCR-induced cytokine production in both cell types. Efforts are underway to assess the relative impact of LT on NKT cells as compared to other immune cell types. Regardless of whether anthrax LT affects one immune cell type more than another the sustained deleterious effects of toxins on B cells, DC, Th and NKT cells is likely to suppress the initiation of the adaptive immune response and reduce the ability of the host to clear the pathogen. Further study will focus on the implications of an LT insult on subsequent NKT adaptive immune responses to a pathogen challenge.

## Supporting Information

Figure S1 Effect of LT on splenic weight, cell count and NKT frequency. C57BL/6 mice were treated with 100 µg of LT in PBS by the *i.v.* route or mock-treated with PBS alone. (A) After 4 d, spleens were obtained and weighed before isolation of splenocytes which were then enumerated. (B) In a separate experiment, splenocytes were incubated with FcR-blocking mAb 2.4G2 in the presence of α-GC/CD1d tetramer and anti-TCRβ mAb and analyzed by flow cytometry.
(0.10 MB TIF)

Figure S2 Down regulation of NKG2D expression is NKT specific and requires active toxin. (A) C57BL/6 and Jα18^−/−^ mice were treated with 100 µg of LT in PBS by the *i.v.* route or mock-treated with PBS alone. After 4 d, splenocytes were obtained and incubated with FcR-blocking mAb 2.4G2 in the presence of CD1d tetramer, anti-TCRβ and anti-NKG2D mAb (C57BL/6) or anti-NK1.1, anti-TCRβ and anti-NKG2D mAbs (Jα18^−/−^). Cells were then washed, fixed and analyzed by flow cytometry. (B) C57BL/6 mice were treated as in (A) except that LF^H690C^ inactive mutant was administered. Data shows expression of NKG2D by CD1d tetramer^+^/TCRα^+^ cells.
(0.10 MB TIF)

Figure S3 Effect of LT on NKT expression of NKG2D at early and late time points. C57BL/6 mice were treated with 100 µg of LT in PBS by the *i.v.* route or mock-treated with PBS alone. After times indicated (A) splenocytes (B) LN cells and (C) thymocytes were obtained and incubated with FcR-blocking mAb 2.4G2 in the presence of α-GC/CD1d tetramer, anti-TCRβ mAb and anti-NKG2D mAb. Cells were analyzed by flow cytometry.
(0.27 MB TIF)

Figure S4 LT has minimal effect on frequency and number of non-viable NKT cells in isolated splenocyte samples. The same samples described in Figure 3 were re-analyzed this time gating on FSC^lo^/SSC^hi^ cells (density plot, left panel) and then gating on all α-GC/CD1d tetramer-binding cells (dot plot, middle panels) followed by analysis of Annexin V and 7-AAD staining (dot plot, right panels).
(0.17 MB TIF)
