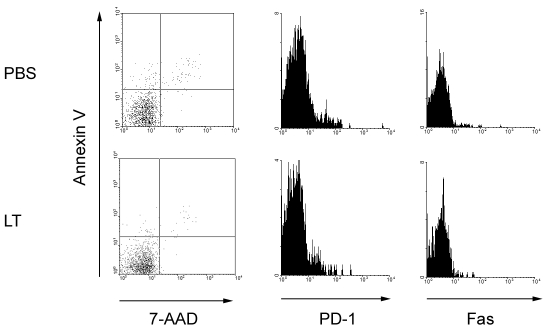# Correction: *Bacillus anthracis* Lethal Toxin Disrupts TCR Signaling in CD1d-Restricted NKT Cells Leading to Functional Anergy

**DOI:** 10.1371/annotation/cfc9c388-05fa-4b1c-8c5f-99835278a458

**Published:** 2009-10-08

**Authors:** Sunil K. Joshi, Gillian A. Lang, Jason L. Larabee, T. Scott Devera, Lindsay M. Aye, Hemangi B. Shah, Jimmy D. Ballard, Mark L. Lang

The flow cytometry dot plots in the left panels of Figure 3 were duplicates. Please see the corrected figure here: 

**Figure:**