# Supplementary figures and images for: Bacillus anthracis Lethal Toxin Disrupts TCR Signaling in CD1d-Restricted NKT Cells Leading to Functional Anergy
Source: PLoS Pathog. 2009 Sep 25;5(9):e1000588. doi: 10.1371/journal.ppat.1000588 (PMC2742733; doi:10.1371/journal.ppat.1000588)

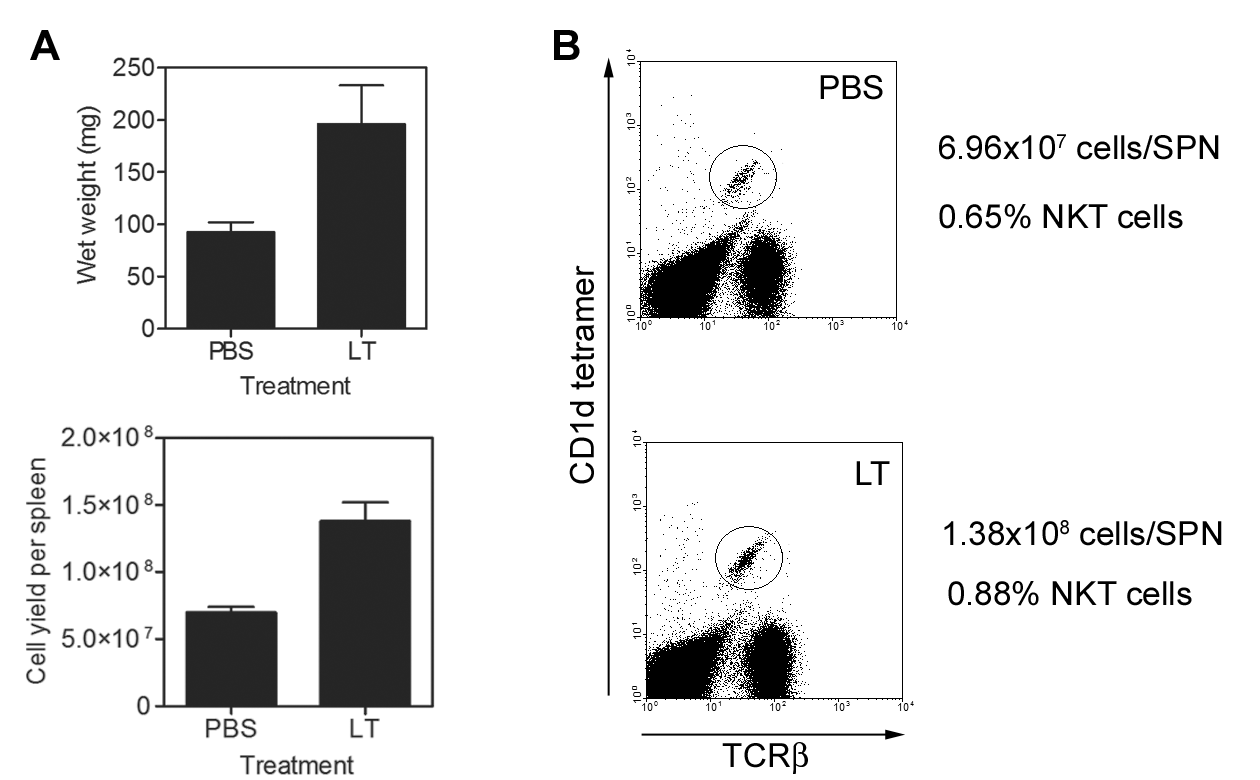

Supplement: Figure S1 — Effect of LT on splenic weight, cell count and NKT frequency. C57BL/6 mice were treated with 100 µg of LT in PBS by the i.v. route or mock-treated with PBS alone. (A) After 4 d, spleens were obtained and weighed before isolation of splenocytes which were then enumerated. (B) In a separate experiment, splenocytes were incubated with FcR-blocking mAb 2.4G2 in the presence of α-GC/CD1d tetramer and anti-TCRβ mAb and analyzed by flow cytometry. (0.10 MB TIF) [file ppat.1000588.s001.tif]

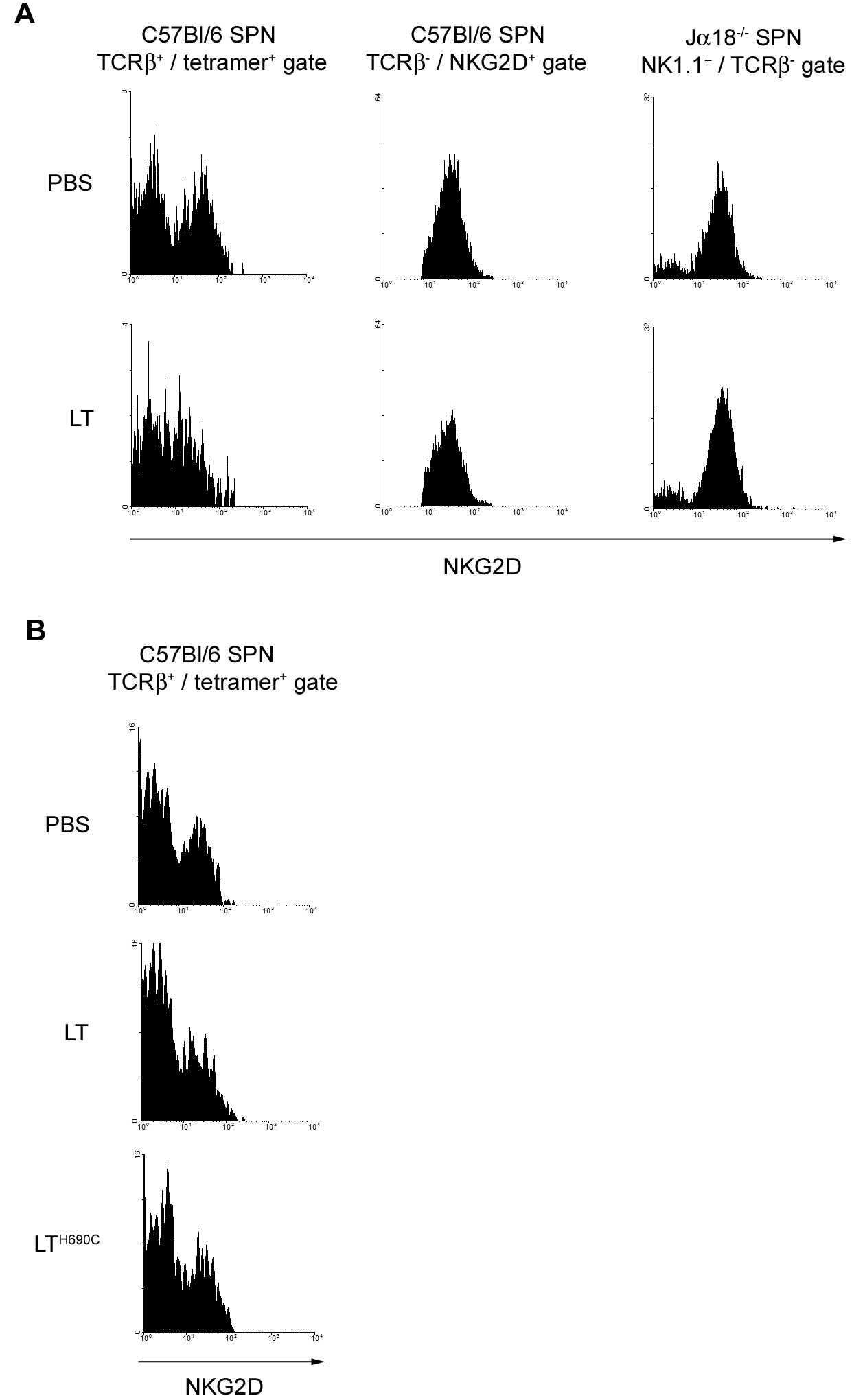

Supplement: Figure S2 — Down regulation of NKG2D expression is NKT specific and requires active toxin. (A) C57BL/6 and Jα18−/− mice were treated with 100 µg of LT in PBS by the i.v. route or mock-treated with PBS alone. After 4 d, splenocytes were obtained and incubated with FcR-blocking mAb 2.4G2 in the presence of CD1d tetramer, anti-TCRβ and anti-NKG2D mAb (C57BL/6) or anti-NK1.1, anti-TCRβ and anti-NKG2D mAbs (Jα18−/−). Cells were then washed, fixed and analyzed by flow cytometry. (B) C57BL/6 mice were treated as in (A) except that LFH690C inactive mutant was administered. Data shows expression of NKG2D by CD1d tetramer+/TCRα+ cells. (0.10 MB TIF) [file ppat.1000588.s002.tif]

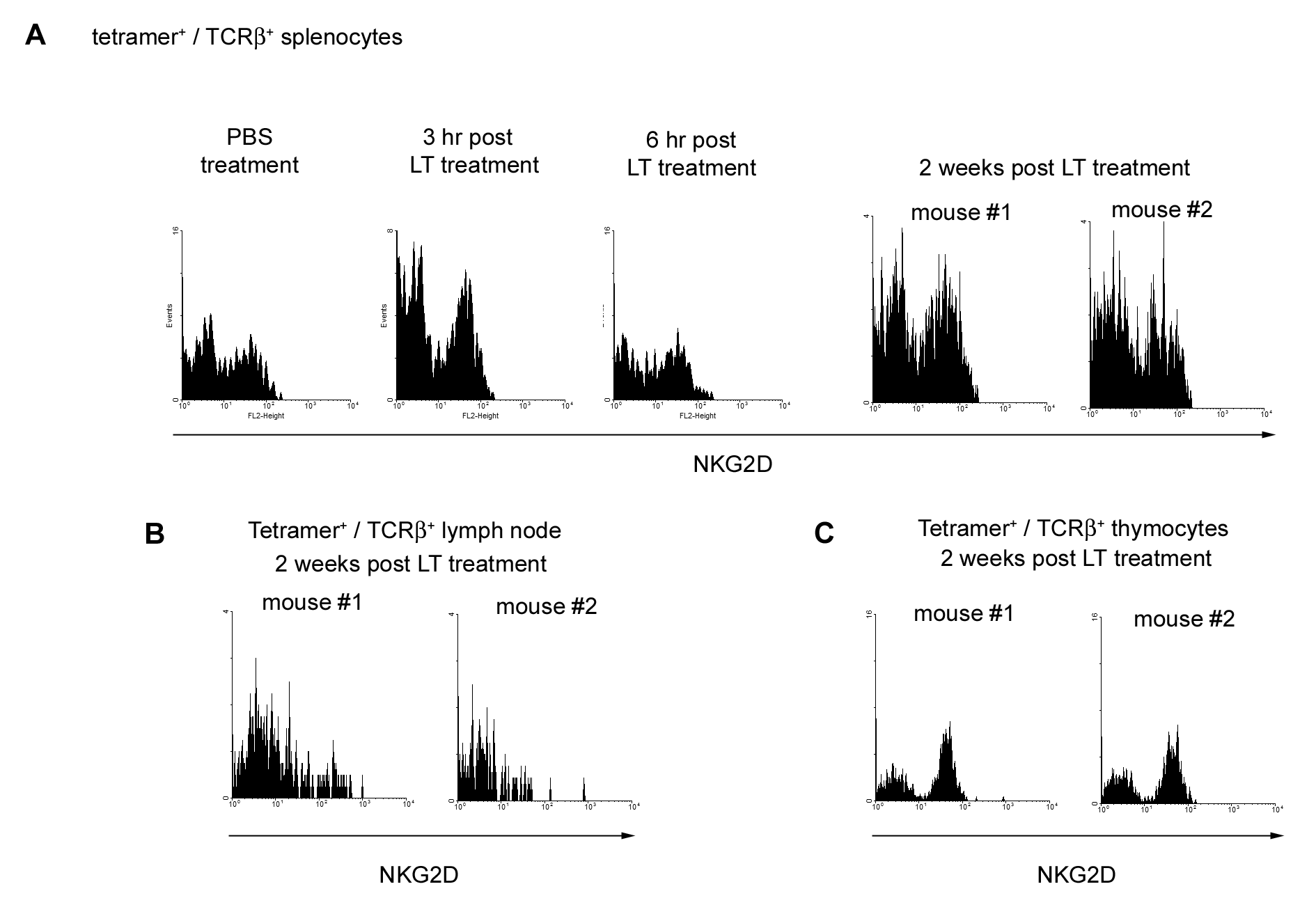

Supplement: Figure S3 — Effect of LT on NKT expression of NKG2D at early and late time points. C57BL/6 mice were treated with 100 µg of LT in PBS by the i.v. route or mock-treated with PBS alone. After times indicated (A) splenocytes (B) LN cells and (C) thymocytes were obtained and incubated with FcR-blocking mAb 2.4G2 in the presence of α-GC/CD1d tetramer, anti-TCRβ mAb and anti-NKG2D mAb. Cells were analyzed by flow cytometry. (0.27 MB TIF) [file ppat.1000588.s003.tif]

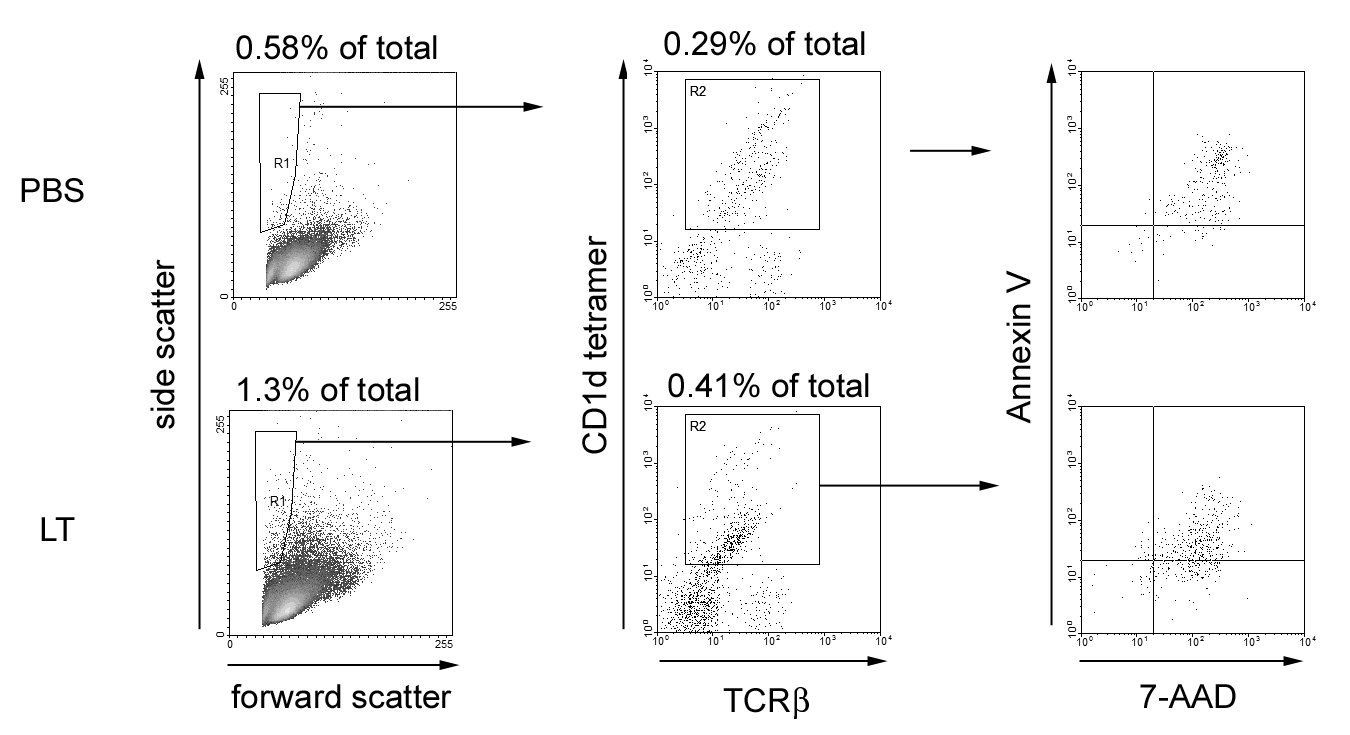

Supplement: Figure S4 — LT has minimal effect on frequency and number of non-viable NKT cells in isolated splenocyte samples. The same samples described in Figure 3 were re-analyzed this time gating on FSClo/SSChi cells (density plot, left panel) and then gating on all α-GC/CD1d tetramer-binding cells (dot plot, middle panels) followed by analysis of Annexin V and 7-AAD staining (dot plot, right panels). (0.17 MB TIF) [file ppat.1000588.s004.tif]
